# Quantitative evaluation of subchondral bone microarchitecture in knee osteoarthritis using 3T MRI

**DOI:** 10.1186/s12891-017-1865-x

**Published:** 2017-11-28

**Authors:** Chenglei Liu, Chang Liu, Xvhua Ren, Liping Si, Hao Shen, Qian Wang, Weiwu Yao

**Affiliations:** 1 Department of Radiology, Shanghai Jiao Tong University Affiliated Sixth People’s Hospital, Shanghai, China; 2 Med-X Research Institute, School of Biomedical Engineering, Shanghai Jiao Tong University, Shanghai, China; 3 Department of Joint Surgery, Shanghai Jiao Tong University Affiliated Sixth People’s Hospital, Shanghai, China

**Keywords:** Magnetic resonance imaging (MRI), Osteoarthritis, Subchondral bone, Knee

## Abstract

**Background:**

Osteoarthritis (OA) is now increasingly recognized as being related to the whole joint instead of the cartilage alone. In particular, the importance of subchondral bone in OA pathogenesis has drawn a lot of interest. The aim of this study is to investigate subchondral bone microstructural features in two femoral condyles of human knee osteoarthritis.

**Methods:**

Eighty subjects were enrolled in our study and divided into three groups: without OA (group 0), mild OA (group 1), and severe OA (group 2). Sagittal 3D Balanced Fast Field Echo (3D–FFE) images were obtained by 3T MRI to quantify trabecular bone structure, and sagittal FatSat 3D Fast Field Echo (3D–FFE) images were acquired to assess cartilage thickness. Trabecular bone parameters, including bone volume fraction (BVF), erosion index (EI) and the trabecular plate-to-rod ratio (SCR), and trabecular thickness were evaluated using digital topological analysis. Subchondral bone and cartilage parameters between different groups and different locations were compared, and their correlations were analyzed.

**Results:**

Within two femoral condyles, subchondral bone structure was deteriorated in mild OA, showing a lower BVF (−0.011 to −0.014 *P* < 0.001), a higher EI (0.346 to 0.310 *P* < 0.001), a lower SCR (−0.581 to −0.542 *P* < 0.001)) and lower trabecular thickness (−6.588 to −4.759 *P* < 0.05). In severe OA, BVF was further decreased, but EI, SCR and trabecular thickness showed no significant difference than mild OA(*P* > 0.05). Moreover, there was a lower BVF, SCR and higher EI in the medial femoral condyle in each group. Interestingly, cartilage attrition mainly occurred in the medial femoral condyle. Medial cartilage thickness was not only positively correlated with the ipsilateral femoral BVF (*r* = 0.321 *P* = 0.004) but also with the opposite femoral BVF (*r* = 0.270 *P* = 0.015).

**Conclusions:**

Our results indicated that deterioration in the trabecular bone structure in both femoral condyles could more sensitively reveal early OA, and BVF could be a better biomarker to evaluate OA severity.

## Background

Osteoarthritis (OA) is a degenerative joint disease that causes joint pain, stiffness and loss of independence. Currently, the structures that initiate disease onset and progression are unclear, and no definitive cure for osteoarthritis is available. The only treatment options include pain control and ultimately joint replacement [1]. Generally, the hallmark of OA has been accepted as articular cartilage loss, and the involvement of subchondral bone was secondary to cartilage attrition. However, recent findings showed that changes in subchondral bone appeared in early-stage osteoarthritis, preceding cartilage deterioration [2]. In models of rat OA, an attenuated subchondral bone plate, decreased trabecular thickness and increased trabecular separation were detected using micro-CT as early as 2 weeks after inducing OA [3, 4]. MRI studies for osteoarthritic knees have also shown that subchondral bone marrow edema-like lesions (BMLs) were associated with pain and increased cartilage loss in the same region in early OA [5].

Moreover, subchondral bone and cartilage form a unit in anatomic regions to sustain mechanical forces. When either of these is altered, increased loads will be transmitted from one surface to the other [6]. Increasing evidence suggests that cartilage and subchondral bone interact and that chondrocytes and bone cells are intimately interconnected in the course of disease [7]. On one hand, chondrocytes could transdifferentiate into osteoblasts in the growth plate [8]. On the other hand, various cytokines produced by subchondral bone were able to regulate cartilage metabolism [9]. In addition, pharmacological studies have shown that bone remodeling inhibitor drugs could improve clinical symptoms and reduce structural progression [10, 11]. Therefore, the assessment of bone structure, except for cartilage, is very critical for the elucidation of OA pathogenesis.

Unfortunately, in recent human osteoarthritis studies, most studies have focused on end-stage subchondral bone changes, and these results were mainly derived from analyses of tibia plateaus after joint replacement using micro-CT or histopathology. Most of the knowledge about early subchondral bone changes has come from animal OA models. Moreover, in the knee joint, in contrast to tibia plateaus, more loading forces are concentrated in the two femoral condyles [12]. Very little information is available in the literature on changes in the subchondral bone structure in these locations during OA. In recent years, with improved MRI resolution and post-processing algorithms, a few relevant studies have been performed, but the results failed to reach a conclusion. The initial MRI study demonstrated significant variations in the trabecular bone structure of the femur and tibia based on very few subjects [12]. Afterwards, Chiba K et al. noted that with OA progression, the trabecular bone structure showed osteoporotic changes in the lateral joint and sclerotic changes in the medial joint [13]. Recently, Chang G et al. used 7 T MRI to display deterioration in the subchondral bone microstructure of the distal femur in patients with mild OA [14]. However, in clinical practice, 7 T MRI is not commonly available, and its application is limited due to increased chemical shift variations, radiofrequency power deposition and magnetic field inhomogeneity [15]. Given this, we used 3T MRI to perform a cross-sectional quantitative analysis of the trabecular bone structure and cartilage to further investigate alterations in the subchondral bone microstructure during the course of OA.

## Methods

### Subjects

Our prospective study was approved by the hospital institutional review board (Shanghai No. 6 People’s Hospital), and written informed consent was obtained from all patients. Between September 2016 and march 2017, 80 subjects were recruited by one orthopedic surgeon with 15 years of experience (SH), and diagnosis was based on a clinical examination and an anterior-posterior weight-bearing knee radiograph. The OA subjects were further divided into two groups based on the Kellgrene-Lawrence (KL) classification [16]. Thirty-three patients with KL scores of 1–2 were classified as mild OA (group 1) (17men and 16 women, age = 45.64 ± 9.09 years, body mass index (BMI) = 24.51 ± 3.32 kg/m^2^). Sixteen patients with KL scores of 3–4 were categorized as severe OA (group 2) (7 men and 9 women, age = 58.19 ± 6.34 years, BMI = 24.71 ± 3.19 kg/m^2^). In addition, thirty-one healthy subjects without knee impairment or radiographic signs of OA were classified as control subjects (group 0) (11 men and 20 women, age = 30.23 ± 9.34 years, BMI = 23.51 ± 1.22 kg/m^2^). All subjects were performed a standardized WOMAC questionnaire (Western Ontario and McMaster Universities Arthritis Index) of pain, functional impairment and stiffness [17]. The detailed clinical data for both control and OA groups were summarized in Table 1. We excluded patients with a history of obesity, knee injury or surgery, inflammatory arthritis, osteonecrosis, or other disease that affects bone structure.

**Table 1:** Clinical data for the three subject groups divided according to the degree of osteoarthritis (OA)

	Total (*n* = 80)	Control (*n* = 31)	Mild OA(*n* = 33)	Severe OA(*n* = 16)	*P* value
Age	42.18 ± 13.68	30.23 ± 9.34	45.64 ± 9.09†	58.19 ± 6.34†	0.000*
Gender					
Male	35(43.75%)	11(35.48%)	17(51.51%)	7(43.75%)	0.434
Female	45(56.25%)	20(64.52%)	16(48.49%)	9(56.25%)	
Knee joint					
Left	30(37.50%)	8(25.81%)	16(48.49%)	6(37.5%)	0.173
Right	50(62.50%)	23(74.19%)	17(51.51%)	10(62.5%)	
weight	66.01 ± 9.56	63.94 ± 6.39	68.03 ± 11.73	65.88 ± 9.38	0.233
BMI	24.17 ± 2.69	23.51 ± 1.22	24.51 ± 3.32	24.71 ± 3.19	0.222
WOMAC OA index					
Pain	4.84 ± 5.39	0.00 ± 0.00	5.30 ± 2.76	13.25 ± 3.55	0.000*
stiffness	0.96 ± 1.97	0.00 ± 0.00	1.00 ± 1.71	2.75 ± 3.00	0.000*
Function	9.16 ± 12.57	0.00 ± 0.00	9.61 ± 9.29	26.00 ± 13.14	0.000*

Data compared with ANOVA for continuous variables, Chi-squared test for categorical variables

*Statistical significance, *p* < 0.05

†Significantly different compared with normal group as determined using Scheffe’s test

### Imaging modality

Radiographs of the knee were performed on a DR (Siemens, Germany), and all patients were asked to stand with the patella facing forward in weight-bearing position while a standard plain X-ray in the anteroposterior plane was taken.

MR imaging of the knee (affected knee in OA subjects, nondominant knee in control subjects) was performed on a 3.0-T superconducting MR scanner (Koninklijke Philips NV, Amsterdam, the Netherlands) with an eight-channel knee coil. The knee flexion angle was adjusted 15°, and a dedicated holder was used to reduce motion artifacts at the time of imaging. The MR imaging protocol included two pulse sequences. The imaging sequences and parameters of each examination were as follows: Sagittal 3D Balanced Fast Field Echo Sequence (3D B-FFE) (TR/TE 19/9.7, FOV 10 cm, matrix 558 × 554, 80 slices, section thickness 1 mm, flip angle 40°, acquisition time 9 min 18 s, in-plane spatial resolution 0.18 × 0.18 mm^2^) to image the subchondral bone microarchitecture, and Sagittal FatSat3D Fast Field Echo Sequence (3D–FFE) (TR/TE 25/4.8, FOV 15 cm, matrix 500 × 499, 80 slices, section thickness 1 mm, flip angle 30°, acquisition time 9 min 12 s, in-plane spatial resolution 0.30 × 0.30 mm^2^) to image the cartilage.

### Image analysis

**Fig. 1:**
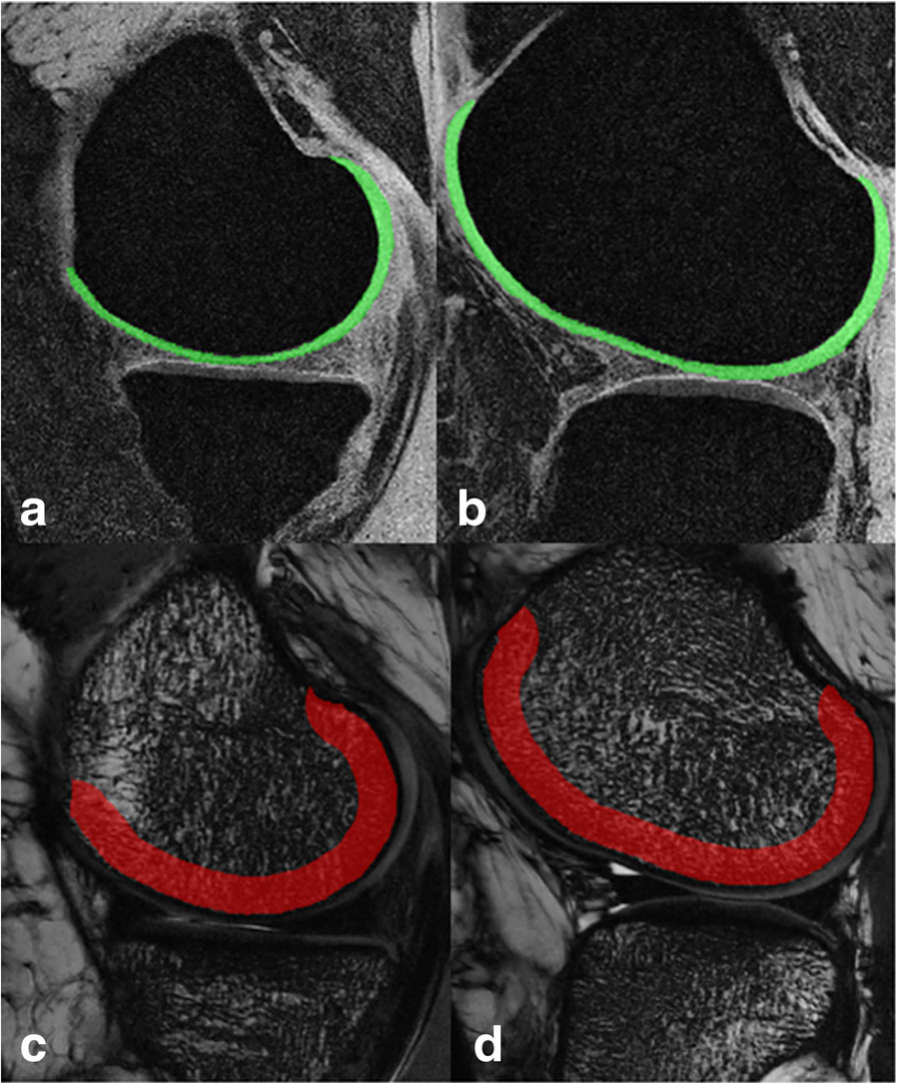
Images of the regions of interest acquired by 3T MRI. Analysis of the cartilage region in the center of the medial femoral condyle (**a**) and the lateral femoral condyle (**b**) by 3D FFE imaging and the analysis of the subchondral bone region in the center of the medial femoral condyle (**c**) and the lateral femoral condyle (**d**) by 3D BFFE imaging

A 3D measurement of the subchondral bone microstructure and cartilage was performed in the sagittal plane using an in-house program created with MATLAB (Math Works, Natick, MA). Five successive slices within the center of the medial and lateral joints were separately selected by a musculoskeletal radiologist (CLL). Prior to the quantitative analysis of the subchondral bone microstructure, N4 correction and a non-local means denoising approach were applied to correct signal intensity variations and image artifacts [18]. The region of interest (ROI) for subchondral bone was 5 mm wide below the subchondral bone plate covered by cartilage in each slice (Fig. 1). The ROI was established semi-automatically by a single biomedical engineer (CL). Then, the 3D interpolations function and the local thresholding algorithm were used to yield a 3D binary volume. For each VOI, a bone volume fraction (BVF) map was created by scaling voxel signal intensities from 0 to 100 (0 = pure marrow, 100 = pure bone). We utilized the digital topological analysis (DTA) to determine subchondral bone networks and compute topological classes for each ROI [19]. The measured parameters included BVF, trabecular network osteoclastic resorption (erosion index, EI), and the trabecular plate-to-rod ratio (SCR). The Fuzzy distance transform method was used for trabecular thickness measurements [20]. The cartilage was segmented using ITK-SNAP. The radiologist (CLL) manually delineated the cartilage using a graphics cursor. The cartilage region of each femoral condyle corresponded to the ROI for the trabecular bone analysis, and cartilage thickness was separately calculated (Fig. 1).

### Statistical analysis

A statistical software package (SPSS 16.0, SPSS, Chicago, III) was used to perform the statistical analysis. The mean value and standard deviation were computed for each trabecular parameter and cartilage thickness in each femoral condyle. The significance of the observed differences between the medial and lateral femoral condyles was established using Student’s paired *t* test. The subchondral bone and cartilage parameters between the groups were compared using an analysis of covariance (ANCOVA) with an adjustment for age. The correlation between cartilage thickness and subchondral bone parameters were analyzed by Pearson’s correlation coefficient test; *P* < 0.05 was considered statistically significant.

## Results

### Trabecular bone structure and cartilage image processing

**Fig. 2:**
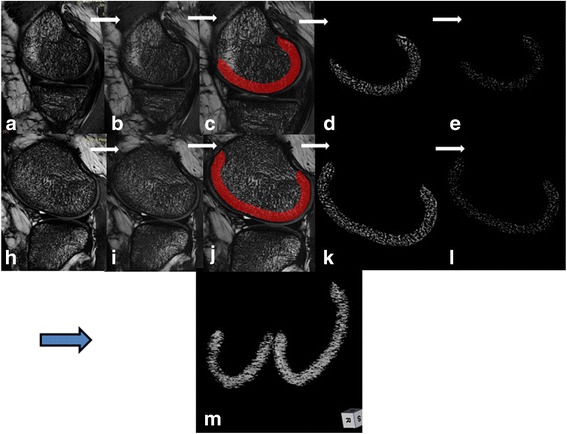
Image processing steps. 1). In vivo raw MR sagittal images in the center of the medial knee joint (**a**) and the lateral knee joint (**h**). 2). Correction of signal intensity variations and image artifacts (**b**, **i**). 3). Establishment of the ROI of subchondral bone (**c**, **j**). 4). Generation of the bone volume fraction map of a single slice (**d**, **k**). 5). Image thinning for the topological analysis (**e**, **l**). 6) Generation of a 3D BVF map (**m**)

**Fig. 3:**
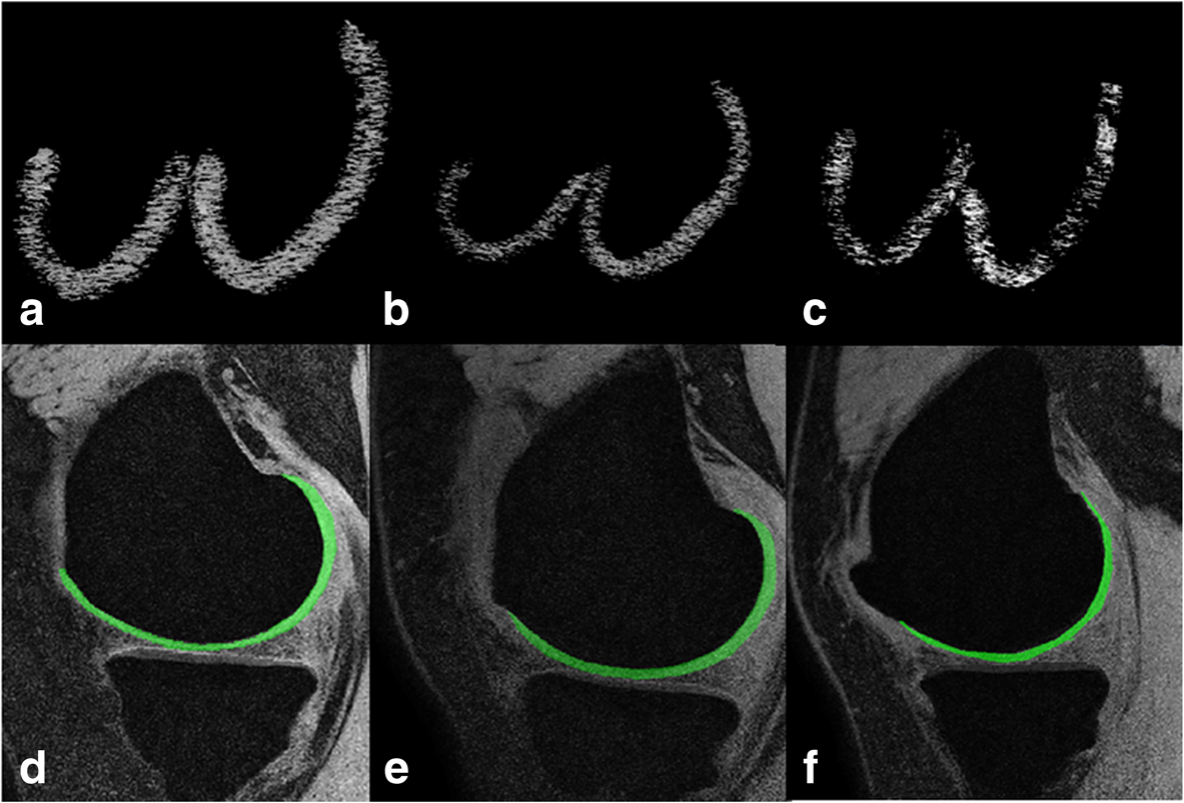
Representative images of the subchondral bone and cartilage between varying stages of OA. For the analysis of the trabecular bone microstructure (**a**-**c**), compared to healthy control subjects (**a**), microstructural deterioration was observed in the medial and lateral femoral condyles of patients with mild OA (**b**). With disease progression, the degree of deterioration was further aggravated (**c**). For the analysis of the cartilage, there were no significant changes in cartilage thickness or volume in the medial condyle in mild OA subjects (**e**) compared with control subjects (**d**), but in the patients with severe OA (**f**), cartilage thickness was significant thinner than in mild OA and control subjects

The data processing steps for the trabecular bone analysis are shown in Fig. 2. MR image intensity inhomogeneity was corrected and image artifacts were dropped. The MR image and BVF map clearly depict trabecular orientation and spatial distribution in the medial and lateral femoral condyles. Representative 3D BVF maps for the subchondral bone microstructures of control subjects, mild OA subjects and severe OA subjects are shown in Fig. 3 (a-c). Compared to the control subjects, the osteoarthritis subjects demonstrated sparse trabeculae and heterogeneous distribution in both femoral condyles and as the degree of OA increased, deterioration in the trabecular bone microarchitecture became further aggravated.

Representative cartilage segmentation images for varying degrees of OA are shown in Fig. 3 (d-f). There was an elaborate fitting in the inner and outer boundaries of the cartilage. Within the medial femoral condyle, compared to the control subjects, cartilage thickness did not significantly change in mild OA subjects. However, in severe OA, the cartilage was obviously thinning.

### Quantitative analysis of topological parameters in the subchondral bone structure and cartilage thickness

#### Medial-lateral differences of trabecular structure and cartilage thickness in the femoral condyles

The descriptive statistics for bone and cartilage measurements by group were summarized in Table 2, and the mean medial-lateral differences in the femoral condyle for each group were shown in Table 3. For each group, significant differences were observed in the BVF, EI and SCR between medial and lateral femoral condyle. The BVF and SCR values were higher and the EI was lower in the lateral femoral condyle. However, no significant difference was observed in trabecular thickness between both femoral condyles. For cartilage thickness, no significant difference was observed between the two femoral condyles in the control subjects, but in the mild OA and severe OA subjects, the differences arrived at statistical significance (*P* < 0,05). Cartilage attrition mainly occurred in area that lower bone topological parameter (medial femoral condyle), which was an interesting finding.

**Table 2:** Mean bone and cartilage parameter values for both femoral condyles between different groups of subjects

	Control (*n* = 31)	Mild OA(*n* = 33)	Severe OA(*n* = 16)
Medial femoral condyle			
BVF	0.145 ± 0.002	0.129 ± 0.002^a^	0.115 ± 0.003^a, b^
EI	2.086 ± 0.060	2.433 ± 0.045^a^	2.599 ± 0.082^a^
SCR	5.842 ± 0.115	5.261 ± 0.087^a^	5.168 ± 0.158^a^
Trabecular Thickness(um)	174.412 ± 1.690	167.823 ± 1.278^a^	170.817 ± 2.315
Cartilage Thickness (mm)	1.361 ± 0.051	1.199 ± 0.038	0.789 ± 0.069^a, b^
Lateral femoral condyle			
BVF	0.154 ± 0.002	0.139 ± 0.002^a^	0.124 ± 0.003^a,b^
EI	1.985 ± 0.054	2.296 ± 0.041^a^	2.478 ± 0.074^a^
SCR	5.531 ± 0.120	4.989 ± 0.091^a^	4.935 ± 0.164^a^
Trabecular Thickness(um)	173.337 ± 1.381	168.578 ± 1.044^a^	169.695 ± 1.892
Cartilage Thickness (mm)	1.365 ± 0.053	1.315 ± 0.041	1.207 ± 0.073

Data are mean ± standard error. The significance between groups is shown based on ANCOVA with adjustment for age

^a^ *P* < 0.01 in comparison with control subjects

^b^ *p* < 0.01 in comparison between mild OA and severe OA

**Table 3:** Mean medial - lateral differences in cartilage and bone parameter values between different groups of subjects

Group	BVF	EI	SCR	Trabecular Thickness(um)	Cartilage Thickness (mm)
0(*n* = 31)	−0.011 ± 0.007**	0.099 ± 0.047**	0.322 ± 0.149**	1.481 ± 4.661	−0.058 ± 0.161
1(*n* = 33)	−0.010 ± 0.010**	0.137 ± 0.104**	0.268 ± 0.197**	−0.872 ± 6.726	−0.100 ± 0.137**
2(*n* = 16)	−0.007 ± 0.011*	0.122 ± 0.101**	0.217 ± 0.316*	0.576 ± 9.706	−0.345 ± 0.224**

Data are mean ± standard deviation. The Mean medial - lateral differences in cartilage and bone parameter values between different groups of subjects is shown based on Student’s paired *t* test. * = *p* < 0.05, ** = *P* < 0.001. Group0 = control subjects, Group1 = mild OA subjects, Group2 = severe OA subjects

#### Differences in trabecular structure and cartilage thickness between controls and patients with OA

**Fig. 4:**
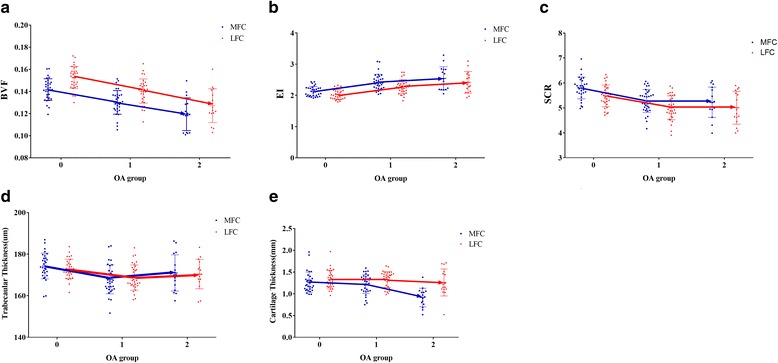
The trends in subchondral bone parameters and cartilage thickness for the medial and lateral femoral condyles between different OA subjects. Group 0 = healthy control subjects; Group 1 = patients with mild OA; Group 2 = patients with severe OA. BVF = bone volume fraction. EI = Erosion Index. SCR = trabecular plate-to-rod ratio. MFC = Medial femoral condyle, LFC = lateral femoral condyle

The trends in subchondral bone parameters and cartilage thickness in both femoral condyles between different OA subjects are shown in Fig. 4. As shown in Fig. 4, within the medial femoral condyle, compared to control subjects, the BVF, SCR and trabecular thickness showed marked decreases, and the EI rise sharply in the mild OA subjects. In the severe OA subjects, the BVF appeared to decrease further, the EI increased slightly, the SCR decreased slightly, and trabecular thickness increased slightly. Within the lateral femoral condyle, a similar trend was apparent during the course of OA. Within the medial femoral condyle, compared to control subjects, cartilage thickness slightly decreased in mild OA subjects. However, in severe OA, the cartilage markedly decreased. Within the lateral femoral condyle, cartilage thickness only slightly decreased during OA.

To evaluate the magnitude of these differences, the absolute value of each parameter for each OA group in the medial and lateral femoral condyles was calculated and summarized into Table 4. As illustrated in Table 4, in the comparison of control subjects and mild OA subjects, the absolute values for the BVF, EI, SCR and trabecular thickness showed significant differences in both the medial and lateral femoral condyles (*P* < 0.05). In the comparison of control subjects and severe OA subjects, significant differences were also seen in each parameter, except for trabecular thickness (*P* < 0.05). However, in the comparison of mild OA and severe OA subjects, a significant difference was seen only in BVF, and the other parameters did not show significant differences. For cartilage thickness, no significant difference was observed between the control subjects and mild OA subjects in the medial femoral condyle. However, in the comparison of mild OA and severe OA subjects, a significant difference was seen. Within the lateral femoral condyle, cartilage thickness only slightly decreased, but a significant difference was not found between the different OA groups.

**Table 4:** Mean differences in the cartilage and bone parameters for both femoral condyles between different groups of subjects

Region, group	BVF	EI	SCR	Trabecular Thickness(um)	Cartilage Thickness(mm)
MFC					
0 and 1	0.011 ± 0.003*	−0.346 ± 0.081*	0.581 ± 0.156*	6.588 ± 2.281*	0.161 ± 0.068
1 and 2	0.014 ± 1.06*	−0.166 ± 0.087	0.092 ± 0.168	−2.993 ± 2.457	0.411 ± 0.073*
0 and 2	0.029 ± 0.005*	−0.512 ± 0.120*	0.673 ± 0.232*	3.595 ± 3.392	0.571 ± 0.101*
LFC					
0 and 1	0.014 ± 0.003*	−0.310 ± 0.073*	0.542 ± 0.162*	4.759 ± 1.864*	−0.050 ± 0.071
1 and 2	0.015 ± 0.004*	−0.182 ± 0.078	−0.053 ± 0.175	−1.117 ± 2.009	0.108 ± 0.077
0 and 2	0.029 ± 0.005*	−0.492 ± 0.108*	0.596 ± 0.241*	3.642 ± 2.773	0.158 ± 0.107

Data are mean ± standard error The significance between groups is shown based on ANCOVA with adjustment for age. The differences are based on group0-group1, group1-group2 and group0-group2. * = *p* < 0.05. Group0 = control subjects, Group1 = mild OA subjects, Group2 = severe OA subjects. MFC = medial femoral condyle, LFC = lateral femoral condyle

#### Correlations between the subchondral bone parameters and cartilage thickness

**Fig. 5:**
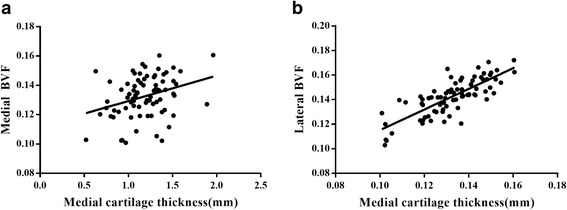
Scatterplot showing relationship between medial femoral condyle cartilage thickness and both medial and lateral BVF. **a** Medial femoral condyle cartilage thickness positively correlated with medial BVF (*r* = 0.321 *P* = 0.004) .**b** Medial femoral condyle cartilage thickness also positively correlated with lateral BVF((*r* = 0.270, *P* = 0.15)

To further identify the dynamics between cartilage degeneration and subchondral bone structure, we investigated the interrelationship between trabecular topological parameters and cartilage thickness in both femoral condyles. As shown in Fig. 5, medial cartilage thickness was not only positively correlated with the medial BVF (*r* = 0.321 *P* = 0.004) but also positively correlated with the lateral BVF (*r* = 0.270, *P* = 0.15). Correlations between other trabecular topological parameters and medial cartilage thickness were not found. Moreover, the relationships between lateral cartilage thickness and trabecular parameters showed no significant correlations (*P* > 0.05).

## Discussion

Our current findings showed that within two femoral condyles, subchondral bone structure was deteriorated in mild OA, and in the severe OA, osteoporotic changes in the trabecular bone were further aggravated. Moreover, the trabecular bone structure showed significant variations in the medial and lateral femoral condyles. Cartilage attrition mainly occurred in area that lower bone topological parameter (medial femoral condyle). In addition, our findings further confirmed that cartilage degeneration was accompanied by changes in the subchondral bone structure.

In mild OA, our findings showed that the BVF, SCR, and trabecular thickness significantly decreased and the EI significantly increased, indicating subchondral bone quality deterioration. These results were consistent with those of prior animal OA studies that showed subchondral bone plate thinning, increased porosity and BVF loss in early OA [21, 22]. In contrast, cartilage thickness loss was not significant, indicating that deterioration of the trabecular bone structure may more easily reveal early OA. Moreover, our finding was also similar to that of Chang G et al. reported using 7 T MRI. Although the spatial resolution of 3T MRI was limited, we came to consistent conclusions relative to trabecular structure changes in mild OA. In addition, in our study, sample size and disease stages increased, sagittal imaging was performed to evaluate subchondral bone and cartilage simultaneously. Increasing evidences showed that weaker subchondral bone quality alters stress-distributing and load-absorbing on cartilage, causing physical damage to the cartilage. In turn, cartilage loss also increases biomechanical loading on the subchondral bone plate and promotes subchondral bone remodeling [23, 24].

In severe OA, the BVF further decreased in both femoral condyles, which was inconsistent with previous studies that noted bone sclerosis with an increased BVF in advanced OA. These results may be due to subchondral bone cyst formation and disuse atrophy in both femoral condyles. Subchondral cysts represent a process of osteoclast-mediated bone resorption, showing decreased trabecular bone mineralization with interruptions and holes [25]. In addition, in the end-stage of OA, deceased activity may also cause osteoporotic changes in the trabecular bone structure. However, compared with mild OA, the EI slightly increased and trabecular thickness was greater, which may indicate that trabecular resorption slowed down. These findings may be related to subchondral bone marrow edema-like lesions (BMLs) and bone formation. Studies have shown that BMLs reflected increased bone remodeling that corresponded to trabecular bone microfractures and bone marrow fibrosis. BMLs showed higher BVF values, trabecular numbers, and thickness [26, 27]. In advanced OA, the subchondral bone microstructure was relatively heterogeneous and microscopic changes varied across the joint surface. These may partially account for this phenomenon. Using synchrotron radiation computed tomography imaging in end-stage OA, Chiba et al. also showed bone sclerosis and bone cysts mixed in the subchondral bone microstructure [25].

Moreover, our study found higher BVF, SCR and lower EI in the lateral femoral condyle when compared with the medial condyle from same group, which may be due to differences in anatomical structures and loading functions. On the sagittal plane, the lateral condyle had a wider loading surface and the loading force was more concentrated in the lateral condyle [12]. Wolff J also stated that subchondral bone remolding depends on the forces placed upon it [28]. Interestingly, cartilage attrition mainly occurred in areas that lower BVF and SCR (medial femoral condyle). This indicated cartilage degeneration was influenced by the remodeling of the underlying subchondral bone. In the future, the prevention of OA progression may be achieved through modifications of biomechanical loading and gait.

Furthermore, our findings further confirmed that medial cartilage degeneration was not accompanied by deterioration in the ipsilateral compartment but with loss in the opposite compartment. As the medial cartilage thickness loss, in the medial femoral condyle, BVF decreased, which may be due to a loading force shift from femur to tibia. Ding and Bobinac et al., using micro-CT reported BVF and trabecular thickness increased in the medial tibia and showed bone sclerosis in knee OA patients [29, 30]. In the lateral femoral condyle, BVF also decreased, which may be attributed to loading force from the lateral joint to the medial joint. Compared with BVF, correlations between other trabecular topological parameters and cartilage thickness loss were not found, which indicated BVF may be a more sensitive biomarker for evaluating OA severity. In the future, it may be used to monitor the efficacy of an osteoarthritis intervention.

In this study, we focused on a digital topological analysis, which has been validated and has shown its relative immunity to partial volume blurring and noise [31]. It has been used to quantify the architecture of human trabecular bone in MR images acquired from cadavers and in vivo [14, 32]. Our results further validated its availability and practicability.

There are several potential limitations in our study. First, lack of age-matched control groups was our primary limitation. Second, in our study, we acquired images in the sagittal direction to evaluate subchondral bone and cartilage simultaneously. This may have increased the partial volume effect in slice dimensions. Third, we conducted a cross-sectional study based on a relatively small number of subjects, so a potential selection bias or unknown confounding factors may be present. In the future, we will perform a longitudinal study of OA subjects to further investigate the temporal sequence of changes in the subchondral bone microstructure and cartilage. Finally, the image acquisition and data processing time was relatively long. Image processing and analysis algorithms should be further optimized.

## Conclusions

In conclusion, the present results indicated that, with limited spatial resolution, 3T MRI coupled with image processing could simultaneously evaluate the three-dimensional trabecular bone microarchitecture and cartilage morphology in living patients with OA. In the mild OA subchondral bone microstructure was deteriorated in both femoral condyles, and as increased severity of OA, osteoporotic changes in the subchondral bone were further aggravated. In addition, medial cartilage thickness was positively correlated with both medial BVF and lateral BVF. These results indicated that MRI derived structure parameter could be a potential marker for evaluating OA severity. Overall, our results further confirmed the concept that poor subchondral bone quality was associated with OA, and may serve as a new potential therapeutic target in osteoarthritis. In the future, this technique could be used to monitor the efficacy of treatments targeting the subchondral bone tissue.

## Abbreviations

BMI: Body mass index
BVF: Bone volume fraction
DTA: Digital topological analysis
EI: Erosion index
MRI: Magnetic resonance imaging
OA: Osteoarthritis
ROI: Region of interest
SCR: Trabecular plate-to-rod ratio
